# Enlarged jugular veins

**DOI:** 10.1007/s12471-016-0940-5

**Published:** 2017-01-03

**Authors:** M. Boulaksil, R. M. M. Gevers

**Affiliations:** 1 Department of Cardiology, Jeroen Bosch Hospital, ’s-Hertogenbosch, The Netherlands; 2 Department of Cardiology, Radboud University Medical Center, Nijmegen, The Netherlands

An 83-year-old woman presented to our emergency department with a two-week history of progressive dyspnoea on exertion and leg oedema, and no syncope. She had a history of hypertension, diabetes mellitus type 2, renal insufficiency, and left bundle branch block.

She was clinically mildly decompensated. The ECG showed sinus rhythm with total AV block and a ventricular escape rhythm of 30/min. Echocardiography showed normal left and right ventricular systolic function and a dilated inferior vena cava with decreased variation.

During pacemaker implantation, a remarkable anatomy of the jugular veins was noticed. An aberrant trajectory of the wire was perceived from the left subclavian vein to the contralateral side (Fig. 1a and online video). After contrast injection, two large veins were observed running parallel to one another which were connected caudally (Fig. 1b). Furthermore, the left brachiocephalic vein and superior vena cava (Fig. 1b and 1c) are appreciated.

**Fig. 1:**
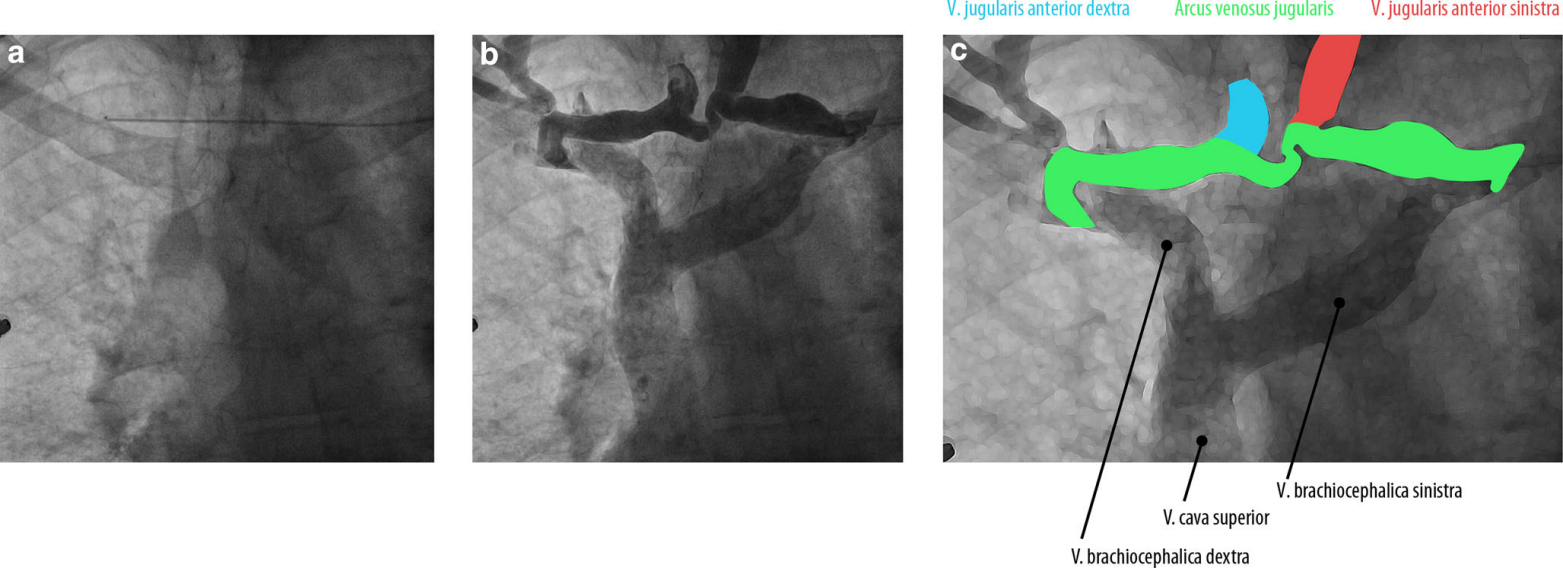
**a** Chest radiography showing a guide wire (arrow head) running horizontally from the left subclavian vein to the contralateral side. **b** An overlay of sequential chest X‑rays showing a phlebogram of two anterior jugular veins running parallel *(venae jugulares anteriores)* which are connected caudally by a jugular arch *(arcus venosus juguli)*. See also the online video

We concluded that the anatomy consisted of enlarged anterior jugular veins *(venae jugulares anteriores)* and a jugular arch *(arcus venosus juguli)* [1]. This is a common anatomy, but these jugular veins are rarely enlarged [2]. Probably, this has to do with the increased venous pressure due to backward heart failure.

## Caption Electronic Supplementary Material

Video of serial contrast injections showing flebograms of two anterior jugular veins (venae jugulares anteriores) running parallel which are connected caudally by a jugular arch (arcus venosus juguli). See also Fig. 1.
